# Microbiological and Pharmacological Evaluation of the Micropropagated *Rubus liebmannii* Medicinal Plant

**DOI:** 10.1155/2012/503031

**Published:** 2012-08-26

**Authors:** Adelina Jiménez-Arellanes, Jorge Cornejo-Garrido, Gabriela Rojas-Bribiesca, María del Pilar Nicasio-Torres, Salvador Said-Fernández, Benito David Mata-Cárdenas, Gloria María Molina-Salinas, Jaime Tortoriello, Mariana Meckes-Fischer

**Affiliations:** ^1^Unidad de Investigación Médica en Farmacología, Hospital de Especialidades, CMNS XXI, Instituto Mexicano del Seguro Social (IMSS), Avenue Cuauhtémoc 330, Col. Doctores, 06720 Mexico City, DF, Mexico; ^2^Centro de Investigación Biomédica del Sur (CIBIS), IMSS, Argentina No. 1, 62790 Xochitepec, MOR, Mexico; ^3^Centro de Investigación Biomédica del Noreste (CIBIN), IMSS, San Luis Potosí y Dos de Abril, Col. Independencia, 64720 Monterrey, NL, Mexico; ^4^Facultad de Medicina, Universidad Autónoma de Nuevo León (UANL), Avnue Fco. I. Madero y E. Aguirre Pequeño, Col. Mitras Centro, Monterrey, NL, Mexico; ^5^Centro de Diagnóstico en Metabolismo Energético y Medicina Mitocondrial, Mexico City, DF, Mexico

## Abstract

*Rubus liebmannii* is an endemic species from Mexico used in traditional medicine primarily to treat dysentery and cough. The *in vitro* activity against *Giardia lamblia* and *Entamoeba histolytica* that produces the ethanolic extract of the aerial parts of the plant led us to expand the pharmacological and phytochemical research of this species. Gastrointestinal disorders including amebiasis remain one of the health problems that need to be addressed and it is of interest to find alternatives that improve their treatment. Also, it is important to emphasize that *R. liebmannii* grows wild in the country and is not found in abundance; therefore, alternatives that avoid overexploitation of the natural resource are mandatory. Ongoing with the evaluation of the potentialities that *R. liebmannii* possesses for treating infectious gastrointestinal diseases, the aim of the present study was to evaluate the biological effects and the chemical composition of the micropropagated plant.

## 1. Introduction

*Rubus *genus (Rosaceae) comprises ca. 250 species distributed worldwide. In Mexico, 28 species have been identified, *Rubus liebmannii* Focke being an endemic species. The plant, known as *citun-zarza* and *tsituni *in the Purépecha language, as well as *tunita del cerro*, *zarzamora*, or *zarza* in Spanish, grows wild in the State of Mexico and in those of Michoacán and Jalisco [1, 2]. The ripe fruits are edible, and, in traditional medicine, the infusion of the leaves is utilized to treat diarrhea, cough, and insomnia. Ethnobotanical data bases also report the employment of the infusion prepared with the sprouts to treat dysentery [3]. A preliminary study reported that the ethanolic extract from the aerial parts of wild *R. liebmannii* possesses antiprotozoal activity against *Giardia lamblia *and* Entamoeba histolytica* with 50% Inhibitory concentration (IC_50_) = 56.12 and 51.43 *μ*g mL^−1^, respectively.The most active compounds isolated were (−)-epicatechin and (+)-catechin, while *β*-sitosterol, squalene, nigaichigoside F1, and 3,4-hydroxybenzoic acid were less active against the tested protozoa [4]. Other species of *Rubus* have exhibited antibacterial activity against *Staphylococcus aureus*, *S. epidermidis*, *Micrococcus luteus*, *Escherichia coli*, *Bacillus subtilis*, and *Candida albicans* [5–9].

In Mexico, gastrointestinal diseases are prominent as a serious health problem, and intestinal amebiasis remains highly frequent [10]. As is well known, currently available drugs for the treatment of these disorders may produce severe side effects and increase the appearance of multidrug-resistant pathogens. *G. lamblia* and *E. histolytica *are two of the most clinically important anaerobic protozoa that cause diarrheal disease worldwide. Recently, giardiasis was included in the “Neglected Disease Initiative,” estimating that 280 million people are infected each year with *G. lamblia *[11].

 On the other hand, tuberculosis affected about one third of the world's population, and its treatment remains a challenge that requires new antitubercular agents due to the emergence of multidrug-resistant and extensively resistant *Mycobacterium tuberculosis *strains. In addition, antimicrobial-resistant bacteria are the causes of numerous clinical problems worldwide. The increase of resistance among pathogens causing nosocomial and community-acquired infections are known to be associated with widespread and overutilization of antibiotics. Infectious diseases caused by resistant microorganism are responsible for increased health costs as well as for high morbidity and mortality, particularly in developing countries. Many bacterial strains are resistant to the actual antibiotics and have increased cost in terms of treatment; for example, *Enterococcus faecalis, Pseudomona aeruginosa*, and others are resistant to tetracyclines, aminoglycosides, and fluoroquinolones [12, 13]. This situation led to the search for novel alternatives, and medicinal plants represent a natural source that requires exploration. Furthermore, the application of biotechnological processes permits harvesting, from micropropagation-generated plants, high raw vegetal material with constant characteristics without producing ecosystem alteration; some protocols were previously designed to obtain high regeneration rates of many plant species *in vitro* [14, 15]. The aim of the present study was to evaluate the pharmacological potential of the micropropagated *R. liebmannii* plant. 

## 2. Methods

### 2.1. Plant Material

*R. liebmannii* plant material for this study was obtained from the Acotzingo Greenhouse in the State of Mexico. The species was identified by Abigail Aguilar, M.Sc., and a voucher specimen (15287) was deposited at the IMSSM Herbarium. 

### 2.2. Micropropagation

The micropropagation protocol was carried out in the South Biomedical Research Center (CIBIS) of the Mexican Institute of Social Security (IMSS). *R. liebmannii* micropropagated plants were obtained from axillary bud explants excised and inoculated in Murashige and Skoog medium supplemented with 30 gL^−1^ sucrose, 8 gL^−1^ agar, 6-Benzylaminopurine (BAP, 3.32 *μ*M), and Indole-3-acetic acid (IAA, 0.57 *μ*M). Multiple shoots were obtained from a singular bud during a 60-day culture period. *In vitro *grown shoots were successfully rooted and acclimatized to greenhouse conditions. Conditions for the plants were gradually changed until an adequate environment for growth directly in the soil was reached (90% survival).

### 2.3. Extract Preparation

The dried powdered aerial parts of the micropropagated plant (1,500 g) were macerated with ethanol (3 × 12 L) at room temperature for 72 h. After filtration, the extract was concentrated to dryness under low pressure at 40°C to obtain 250 g of the crude extract (16.7% w/w).

### 2.4. Chemical Study

 The ethanolic extract (50 g) was fractionated by column chromatography on Si gel (Merck) to obtain 15 primary fractions. All fractions were submitted to antimicrobial and spasmolytic testing. Stigmastan-3,5-diene and *β*-sitosterol were detected in F9 as the major compounds, and F10–F12 rechromatographed on Si gel led to the isolation of 12 secondary fractions (F_A_–F_L_), while the combined fraction (F_D_–F_E_) rendered 45 mg (−) epicatechin and 34 mg nigaichigoside F1 after preparative thin-layer chromatography (TLC) using CHCl_3_-Et_2_O (9 : 1) as mobile phase. Chemical identification of these compounds was performed by comparison of spectroscopic data with those of authentic samples.

### 2.5. Test Organisms

 Microorganisms comprised *Staphylococcus pyogenes* (ATCC 29213), *Enterococcus faecalis* (ATCC 29212), *Escherichia coli* (ATCC 25922), *Proteus mirabilis* (ATCC 43071), *Salmonella typhi* (ATCC 06539), *Mycobacterium tuberculosis* H37Rv (ATCC 27294), *Candida albicans* (ATCC 10231), *Trichophyton mentagrophytes* (ATCC 28185), and *T*. *rubrum* (ATCC 28188). Bacteria were maintained and tested on Trypticase soy agar (TSA, Merck) at 37°C, and yeast and fungi, on Sabouraud 4% dextrose agar (SDA, Merck). *M*. *tuberculosis* H37Rv was cultured in Middlebrook 7H9 broth supplemented with 0.2% glycerol and 10% OADC (Oleic acid-Albumin-Dextrose, and Catalase) enrichment. *Entamoeba histolytica* (HMI-IMSS) and *Trichomonas vaginalis *(GT-13) were grown under axenic conditions in PEHPS medium [16]. *Giardia lamblia *(0989:IMSS) was cultivated in TYI-S-33 medium as modified by Keister [17].

### 2.6. Antiprotozoan Assay

The method to determine the anti-protozoan activity of our preparations was performed as described elsewhere [16, 18]. All protozoa species were incubated at 37°C and subcultivated twice weekly. When each protozoa culture was grown logarithmically, trophozoites were harvested and suspended in 50 *μ*L phosphate-buffered saline, pH 7.4 (PBS) at the following concentrations: *E. histolytica* 20 × 10^3^; *G. lamblia* 20 × 10^4^ cell, and *T. vaginalis* 10 × 10^4^ trophozoites per mL^−1^. These suspensions were inoculated separately in 1 mL screw-capped borosilicate vials (Bellco, Vineland, NJ, USA) containing 1 mL of PEHPS for *E. histolytica *and *T. vaginalis*, or TYI-S-33 for *G. lamblia. E. histolytica* cultures were incubated at 37°C for 72 h, and those of *T. vaginalis* and *G. lamblia,* for 24 h. Vials were added containing 50 *μ*L of various concentrations of the *R. liebmannii* extract of interest (10–0.31 *μ*g mL^−1^). The extract was serially diluted with 100% DMSO in twofold steps. A control containing 50 *μ*L of DMSO was included in each assay. After 48 h of incubation at 37°C, the trophozoites were harvested, washed twice with PBS, and reincubated for 48 h in the corresponding fresh medium. The trophozoites from each vial were counted with a hemacytometer. In each assay, metronidazole and emetine were included as internal controls. Concentrations of these drugs were as follows: emetine (0.7 *μ*g mL^−1^) was used exclusively in the *E. histolytica* assays, and metronidazole at 1.6, 1.0, and 0.5 *μ*g mL^−1^ was used for *E. histolytica, T. vaginalis, and G. lamblia,* respectively. The inhibitory effect of the *R. liebmannii* extract was estimated as the decreased percentage of trophozoite number with respect to nontreated cultures. Corresponding averages and standard deviations (SD) were plotted. The fifty percent inhibitory concentration (IC_50_) of the *R. liebmannii* extract was expressed as the concentration that produced a 50% decrease of trophozoite concentration in each of the three protozoa species. Each determination was performed three times, in triplicate. The effect of metronidazole or emetine was expressed as a diminution percentage with respect to nontreated controls.

### 2.7. Antibacterial Assay

 The extract dissolved in DMSO (Merck) was added to the agar culture medium at concentrations of 1, 2, 4, and 8 mg mL^−1^. Inocula containing 10^7^ colony-forming units (CFU) mL^−1^ for bacteria and 10^6^ CFU mL^−1^ for yeast were applied with a steer replicator, and samples were incubated for 24 h at 37°C. Gentamycin (for bacteria), nystatin (for yeast), and ketoconazole (for fungi) were included as reference standards, and assays were performed by duplicate and repeated twice. Results were expressed as the lowest concentration of the extract that produced complete suppression of the colony growth (minimal inhibitory concentration (MIC)) [19].

The previously described Microdilution alamar blue assay (MABA) was utilized to determine the antimycobacterial effect of the plant extract [20] prepared at an initial concentration of 4 mg mL^−1^ in DMSO (Sigma-Aldrich) and was further diluted in Middlebrook 7H9 broth. The concentration of the EtOH extract and pure compounds tested ranged from 100 to 3.13 *μ*g mL^−1^. Rifampin (Sigma-Aldrich) was used as positive control with concentrations ranging from 2.00 to 0.06 *μ*g mL^−1^.

### 2.8. Relaxant Effect on *In Vitro* Guinea-Pig Ileum

The relaxant effect of the samples (extract, fractions, and pure compounds) was evaluated in the spontaneous contractile response induced by K^+^ in isolated guinea-pig ileum following the methodology described by Meckes [21]. Male guinea pigs weighing 450–600 g were stunned and bled. The distal ileum (10 cm above the ileocecal junction) was removed and placed in a Tyrode solution of milliMolar (mM) composition: NaCl, 136; KCI, 2.68; CaCl_2_, 1.84; MgCl_2_, 0.49; NaH_2_PO_4_, 0.36; NaHCO_3_, 11.10; glucose, 11.10 (pH 7.4). Segments of ileum (1.5 cm) oriented along their longitudinal axis sections were suspended in 10 mL organ baths containing Tyrode solution at 37°C, bubbled with 95% O_2_/5% CO_2_, and at a resting tension of 1 g. After a 60 min equilibration period, isometric contractions induced by high-K^+^ (60 mM) depolarizing Tyrode were recorded through a Grass force-displacement transducer (FTO3C) connected to the Grass-7D polygraph. The composition of Tyrode depolarizing solution was (in mM) as follows: NaCl, 78.68; KCI, 60.00; CaCl_2_, 1.84; MgCl_2_, 0.49; NaH_2_PO_4_, 0.36; NaHCO_3_, 11.10; glucose, 11.10 (pH 7.4). 

### 2.9. Toxicological Assay

The study with animals was performed according to the guidelines of the local Ethics Committee for Experimentation in Animals in Mexico (Ministry of Agriculture, NOM-062-ZOO-1999, Mexico) modified in 2001 and was approved by the Institutional Animal Care and Use Committee. Animals were maintained under standard environmental conditions at 12 h light/dark photoperiods. All animals were supplied with food and water *ad libitum* during the testing period.

Acute toxicity was determined in male and female Balb/c (weight, 22 ± 2.2 g) mice and Sprague-Dawley rats (weight, 220 ± 22.0 g) following the methodology previously described by Lorke [22]. The rodents were randomly divided *per *gender into five groups of three animals each. Group 1 was the control DMSO : H_2_O (3 : 7), and groups 2–5 were treated orally with the extract at 1, 1.6, 2.9, and 5 g kg^−1^. The extract solubilized in DMSO : H_2_O (3 : 7) was administered intragastrically in a volume of not >10 mL kg^−1^, and the general behavior of the treated animals was observed at 1, 2, 4, 6, and 24 h, and daily for 14 days. At the end of the study, the internal organs (lung, kidney, heart, spleen, and liver) were extracted, and a gross pathological observation was performed. The 50% Lethal dose (LD_50_) value was determined by Probit. 

Subacute toxicity was determined following the methodology previously described by OECD TG407 [23]. Twenty-five male Sprague-Dawley rats (weight, 220 ± 22.0 g) were randomly assigned to five groups with five animals per group. The crude extract was administered orally by gavage once daily for 21 days. Groups 1 and 2 received distilled water or the vehicle DMSO : H_2_O (3 : 7), and groups 3–5 were treated with the extract at daily doses of 10, 100, and 1,000 mg kg^−1^. General behavior was observed daily, and the weight of the animals was registered once a week. Animals that died during the test period were analyzed pathologically, and those that survived were examined at the end of the experimentation phase. 

On day 21, the rats were fasted overnight and anesthetized with sodium pentobarbital (56 mg kg^−1^) for blood collection from right ventricle. Heparinized blood samples were taken for determining complete blood count (CBC), red blood cell count (RBC), hemoglobin concentration (HGB), hematocrit (HCT), mean corpuscular volume (MCV), mean corpuscular hemoglobin concentration (MCHC), platelets, white blood cell count (WBC), neutrophils, lymphocytes, monocytes, eosinophils, and basophils. Serum from nonheparinized blood was carefully collected for blood chemistry, and enzyme analyses were conducted to determine glucose (GLU), UREA, creatinine (CREAT), total cholesterol (CHOL), triglycerides (TRI), high-density cholesterol (HDL), low-density cholesterol (LDL), aspartate amino transferase (AST), alanine amino transferase (ALT), alkaline phosphatase (ALP), gamma glutamyl transferase (*γ*GT), uric acid (UrAc), total bilirubin (TB), and conjugated bilirubin (DIRB). Hematological and chemistry analyses were performed employing automated equipment (Coulter T890 and Selectra II, resp.). 

Immediately after blood collection, all animals were sacrificed, internal organs (liver, intestine, spleen, and kidney) were removed and weighed to determine relative organ weight, and gross alterations were observed. The tissues were preserved in 10% formaldehyde solution for histological examination, and tissues of the groups treated with the highest dose of the extract (1.0 g kg^−1^ day^−1^) and their corresponding controls were embedded in paraffin and subjected to hematoxylin-eosin staining. The organs of animals treated with lower doses were histologically examined only when lesions were detected at highest doses.

### 2.10. Statistical Analysis

Results are expressed as mean ± standard error of mean (SEM). Significance differences between controls and experimental groups were assessed by Student *t* test, considering *P* < 0.05 as significant. 

## 3. Results and Discussion

In Mexico, the culture of medicinal plants is a scarce phenomenon, and the current demand for these species has increased notably, a circumstance that forces us to search for options to protect these plants from overcollection. Micropropagation technology from existing meristems allows for continuous production of plants that are genetically identical to wild plants [14]. The present paper describes the biological and chemical properties of micropropagated *R. liebmannii*, correlating the effects with those of the wild species. As a first step, the ethanolic extract from the aerial parts of the micropropagated plant was submitted to biological testing to evaluate antiprotozoan activity (Table 1). The results obtained demonstrated a significant effect against *G. lamblia *(IC_50_ = 11.75 *μ*g mL^−1^), that is, more potent than the effect previously reported for the same extract obtained from the aerial parts of the wild plant (IC_50_ = 56.12 *μ*g mL^−1^) [4]. The growth of *E. histolytica* was inhibited with a notably lower potency (IC_50_ > 100 *μ*g mL^−1^) than the reported effect of the wild plant (IC_50_ = 51.43 *μ*g mL^−1^). By comparing these preliminary results, it is possible to assume that although the micropropagated plant material possesses antiprotozoan properties, biological differences with the wild plant could be found. It is well known that many factors exert an important influence on the biosynthesis of secondary metabolites, such as seasonal collection and environmental and ecological factors (temperature, humidity, altitude, soil, and soil microflora) of areas where the plants grow [14, 15]. Chemical fractionation of the ethanolic extract from the micropropagated plant led to obtaining the polyphenol (−)-epicatechin and the triterpenoid nigaichigoside F1 isolated previously from the wild plant [4]; however, (+)-catechin was not detected in the micropropagated plant extract, probably a factor that could explain, in part, the difference between the species' biological effect. The isolated (−)-epicatechin showed an IC_50_ = 30.2 *μ*g mL^−1^ against *E. histolytica* and an IC_50_ = 31.5 *μ*g mL^−1^ against *G. lamblia* and *T. vaginalis*. Nigaichigoside F1 with IC_50_ = 20.5 *μ*g mL^−1^ against *E. histolytica *was highly potent against *G. lamblia* (IC_50_ = 2.17 *μ*g mL^−1^) and against *T. vaginalis* (IC_50_ = 4.92 *μ*g mL^−1^). The antigiardial activity of (−)-epicatechin was also evaluated *in vivo*, resulting in a 50% effective dose (ED_50_) = 0.072 *μ*mol kg^−1^ in suckling female CD-1 mice [24, 25]. 

Taking into account that *R. liebmannii* is used to treat infectious diseases (gastrointestinal and respiratory ailments) and that the antibacterial and antifungal effects of *R. ulmifolius, R. cordifolius*, and* R. chamaemorus *have been described [5, 7, 8], the crude extract and some of the primary and secondary fractions of the micropropagated *R. liebmannii* were tested for antibacterial, antifungal, and antimycobacterial activities. The extract was active only against *S. aureus *(MIC = 1 mg mL^−1^), and moderate activity was also detected against *T. rubrum *and *T. mentagrophytes *dermatophytes (MIC = 2 mg mL^−1^); nevertheless, pure isolated (−) epicatechin and nigaichigoside F1 were inactive against all microorganisms tested (MIC > 8 mg mL^−1^) (data not shown). The crude extract showed a significative effect against *M. tuberculosis *H37Rv (MIC = 0.1 mg mL^−1^) although the pure compounds isolated [(−) epicatechin and nigaichigoside F1] were inactive against this strain (MIC > 0. 1 mg mL^−1^). 

Contractions induced with high K^+^-depolarizing Tyrode reached a steady level of tension after 40–50 min (*E*_max⁡_ = 100%). Further addition of cumulative concentrations of the plant products produced concentration-dependent inhibition of the sustained tonic contractile response. The potency of the inhibitory effect (IC_50_), calculated by the nonlinear fitting curve, showed F8–F10 to be the most active fractions (6.22–27.66 *μ*g mL^−1^), being F9 as potent as papaverine (6.70 ± 1.31 *μ*M), the well-known antispasmodic agent that was included in this assay as a reference (Table 2). The main compounds in these fractions include stigmastan-3,5-diene and *β*-sitosterol. Although (−) epicatechin was detected in fractions F10–F12, the pure compound was not effective as an antispasmodic agent (IC_50_ > 200 *μ*g mL^−1^).

The ethanol extract submitted to toxicological evaluation showed it to be a nontoxic product. LD_50_ obtained by Lorke procedure was >5 g kg^−1^ when both rodents' species were administered with the extract intragastrically. No death was registered throughout the 14 days of observation, nor did we observe macroscopic physical alteration in liver, kidney, lung, or body weight. 

On the other hand, after administration of repeated doses of the extract (10, 100, and 1,000 mg kg^−1^) for 21 consecutive days, no adverse effects or deaths were detected in the treated groups. Body weights increased in constant fashion in all groups, and nonsignificant variation was observed when comparing these with those of the controls; similarly, organ weight of the treated animals was not affected by the extract (data not shown). Hematological analysis did not show variations when compared to that of the control (Table 3). In clinical blood chemistry analyses, we observed that oral administration of the extract produced no alterations in any determined parameter (Table 4). Regarding hepatic and renal functions, the values of the serum transaminases ALP, AST, and ALT were normal, which permits us to assume that the extract does not produce a hepatic toxic effect; on the other hand, UREA and CREAT also demonstrated normal levels in comparison with the respective controls. 

**Figure 1:**
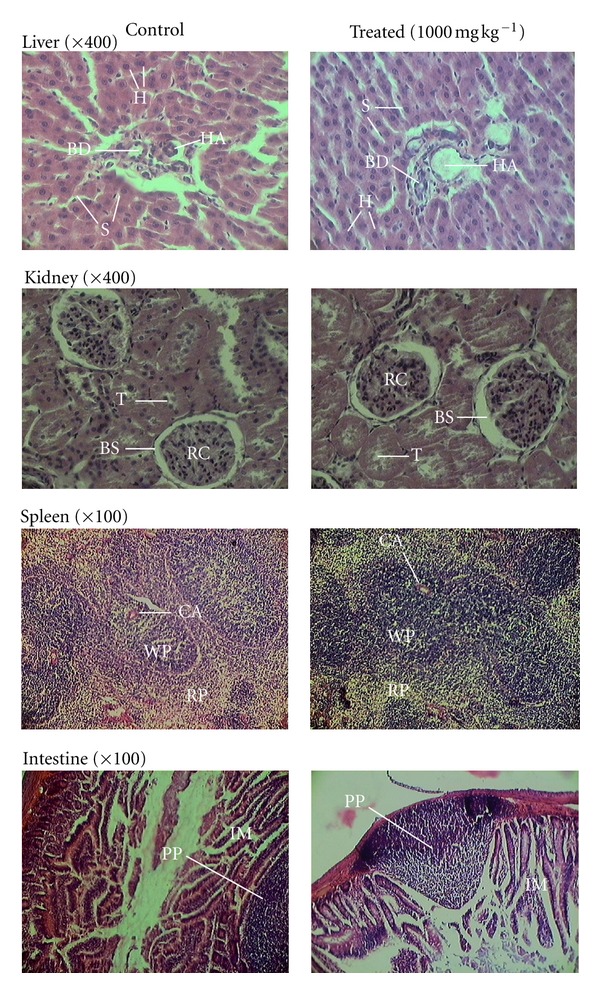
Photomicrographs of liver, kidney, spleen, and intestine from male Sprague-Dawley rats in subacute toxicity of the extract from aerial parts of *Rubus liebmannii.* Cross-sections were stained with hematoxylin and eosin at ×400 (for liver and kidney) and at ×100 (for spleen and intestine). Observations were conducted at different amplified levels. The liver cross-section shows the hepatic artery (HA), bile duct (BD), central vein (CV), sinusoids (S), and hepatocytes (H), all clearly conserved. Cross-section of kidney shows, on magnification, renal corpuscles (RC), tubules (T), and Bowman's space (BS), all sealed. Spleen cross-section shows red pulp (RP) and white pulp (WP) containing central arteries (CAs), all well defined. Cross-section of intestine shows Peyer patches (PPs) and intestinal mucosa (IM), with all of these structures also found to be totally conserved.

At the end of treatment with the extract, histological examination of liver, kidney, spleen, and intestine showed normal architecture, suggesting no morphological disturbances (Figure 1). Renal cortex and renal corpuscles were preserved, as well as tubule types (see Figure 1, kidney).

## 4. Conclusion

The micropropagated *Rubus liebmannii *species is a non-toxic plant with antiprotozoal and antispasmodic properties that may have potential as a therapeutic product. The *in vitro* culture method that has been developed to propagate the species allows the proposal of a clinical trial to demonstrate the safety and efficacy of this plant for treating amebiasis and giardiasis, two gastrointestinal parasitic infections with high prevalence in the majority of developing countries.

## Figures and Tables

**Table 1:** Antiprotozoal activity of ethanol extract and pure compounds from micropropagated* Rubus liebmannii. *

Sample	IC_50_ (*μ*g mL^−1^)		
	*E. histolytica*	*G. lamblia*	*T. vaginalis*
Ethanolic extract	>100	11.75	>100
(−)-Epicatechin	30.2	31.5	31.5
Nigaichigoside F1	20.5	2.17	4.92
Metronidazole	1.6	0.5	1.0

IC_50_: 50% Inhibitory concentration; *E. histolytica: Entamoeba histolytica;*

*G. lamblia: Giardia lamblia; T. vaginalis: Trichomonas vaginalis.*

**Table 2:** Relaxant effect induced by ethanol extract and primary fractions from *Rubus liebmannii* on spontaneous concentration of ileum.

Sample	IC_50_ (*μ*g mL^−1^)
Ethanolic extract	>250
F6	NT
F7	52.99 ± 4.74
F8	27.66 ± 3.41
F9	6.22 ± 0.73
F10	14.87 ± 0.33
F11	>250
F12	34.74 ± 6.81
F13	61.71 ± 9.8
F14	>250
F15	57.50 ± 5.76
*β*-sitosterol	42.81 ± 3.42
(−)-Epicatechin^∗^	>200
Papaverine	14.1 ± 0.35

IC_50_: 50% Inhibitory concentration; NT: Not tested. ^∗^Sigma.

**Table 3:** Hematological values and white blood cell count of male rats in subacute toxicity of the ethanol extract from *Rubus liebmannii. *

Male rats	Vehicle control	Doses (mg kg^−1^)		
		10	100	1,000
RBC × 10^6^ *μ*L^−1^	7.67 ± 0.32	7.67 ± 0.07	8.068 ± 0.07	7.67 ± 0.27
HGB g dL^−1^	14.9 ± 0.65	15.64 ± 0.14	15.98 ± 0.15	15.3 ± 0.26
HCT (%)	43.38 ± 1.20	45.66 ± 0.29	46.36 ± 0.43	44.36 ± 1.10
MCV (fl)	56.5 ± 0.61	59.46 ± 0.25	57.48 ± 0.41	57.86 ± 0.70
MCHC g dL^−1^	34.34 ± 0.22	34.28 ± 0.29	34.48 ± 0.29	34.48 ± 0.37
Platelet (×10^8^ *μ*L^−1^)	7.974 ± 0.78	9.78 ± 0.46	10.56 ± 0.86	10.57 ± 0.67
WBC (×10^3^ *μ*L^−1^)	7.44 ± 1.10	7.2 ± 0.70	10.3 ± 1.48	8.04 ± 2.11
Lymphocyte (%)	81.2 ± 3.13	81.6 ± 1.88	62.8 ± 9.54	71.6 ± 2.50
Neutrophil (%)	18.4 ± 3.32	17.8 ± 2.26	37 ± 9.59	27.8 ± 2.26
Eosinophil (%)	0.8 ± 0.58	0.2 ± 0.2	0.2 ± 0.2	0.4 ± 0.24
Monocyte (%)	0 ± 0	0.4 ± 0.24	0 ± 0	0.2 ± 0.2
Basophil (%)	0 ± 0	0 ± 0	0 ± 0	0 ± 0

RBC: Red blood count; HGB: Hemoglobin; HCT: Hematocrit; MCV: Mean corpuscular volume; MCHC: Mean corpuscular hemoglobin concentration; WBC: White blood count. Results are means ± Standard error of the mean (SEM); *n* = 5; *P* < 0.005.

**Table 4:** Blood chemistry values of male rats in subacute toxicity of the ethanol extract from aerial parts of *Rubus liebmannii. *

Male rats	Vehicle control	Doses (mg kg^−1^)		
		10	100	1000
GLU mg dL^−1^	185.55 ± 11.03	178.42 ± 13.60	175.69 ± 7.39	189.81 ± 20.92
UREA mg dL^−1^	50 ± 3.03	53.40 ± 1.63	51.26 ± 3.61	61.80 ± 1.34
UrAc mg dL^−1^	2.42 ± 0.37	2.15 ± 0.29	1.26 ± 0.18	1.98 ± 0.18
CHOL mmol L^−1^	22.78 ± 1.39	22.14 ± 1.28	21.74 ± 1.61	22.39 ± 1.42
TRI mmol L^−1^	0.72 ± 0.05	0.89 ± 0.14	0.61 ± 0.04	0.78 ± 0.17
HDL mmol L^−1^	5.55 ± 0.17	5.82 ± 0.35	4.36 ± 0.72	5.20 ± 0.17
LDL mmol L^−1^	1.72 ± 0.10	1.73 ± 0.1	1.85 ± 0.30	1.66 ± 0.10
CREAT mg dL^−1^	0.53 ± 0.04	0.58 ± 0.03	0.38 ± 0.06	0.67 ± 0.02
TB mg dL^−1^	0.33 ± 0.03	0.25 ± 0.01	0.25 ± 0.01	0.29 ± 0.03
DIRB mg dL^−1^	0.45 ± 0.08	0.27 ± 0.06	0.22 ± 0.02	0.41 ± 0.10
ALP U L^−1^	605 ± 4.42	602.4 ± 59.27	483.4 ± 52.50	562.4 ± 40.91
AST U L^−1^	105.19 ± 8.12	79.88 ± 2.63	84.28 ± 25.81	163.71 ± 18.10
ALT U L^−1^	63.28 ± 3.35	67.92 ± 3.87	43.59 ± 6.97	82.12 ± 2.13
*γ*GT U L^−1^	0.74 ± 0.05	0.78 ± 0.08	0.52 ± 0.13	0.79 ±0.06

GLU: Glucose; UrAc: Uric acid; CHOL: Total cholesterol; TRI: Triglycerides; HDL: High-density cholesterol; LDL: Low-density cholesterol; CREAT: Creatinine; TB: Total bilirubin; DIRB: Direct bilirubin; ALP: Alkaline phosphatase; *γ*GT: gamma Glutamyl transferase; AST: Aspartate amino transferase; ALT: Alanine amino transferase. Results are means ± Standard error of the mean (SEM); *n* = 5; *P* < 0.005.
